# Supplementary material for: CdS Nanoparticles Supported by Cobalt@Carbon-Derived MOFs for the Improved Adsorption and Photodegradation of Ciprofloxacin
Source: Int J Mol Sci. 2023 Jul 13;24(14):11383. doi: 10.3390/ijms241411383 (PMC10380313; doi:10.3390/ijms241411383)
Supplement: Supplementary file 1 [file ijms-24-11383-s001.zip › ijms-2486138-supplementary.pdf]

Supporting Information

CdS nanoparticles supported by Cobalt@Carbon-derived MOFs for the improved adsorption and photodegradation of Ciprofloxacin

Mi Li[1, 2], Qin Fang[1, 2], Yan Lai[1, 2], Luying Chen[1, 2], Qiucheng Fu[1, 2], Jiao He[1, 2], Yongjuan Chen[1, 2], Liang Jiang[1, 2], Zhiying Yan[1, 2*], Jiaqiang Wang[2, 3*]

1. School of Chemical Sciences & Technology, Yunnan University, Kunming 650091, P. R. China
2. Yunnan Province Engineering Research Center of Photocatalytic Treatment of Industrial Wastewater, Yunnan University, Kunming 650091, P. R. China.
3. School of Materials & Energy, Yunnan University, Kunming 650091, P. R. China.

Preparation of ZIF-8 and ZIF-8@ZIF-67.

ZIF-8 polyhedrons were prepared according to a previously reported method, with some modifications [1]. Briefly, 40 mL of methanol containing 6 mmol of Zn $(NO_3)_2{\cdot}6H_2O$ was added to 20 mL of methanol containing 24 mmol of 2-methylimidazole, and the mixture was aged at room temperature for 24 h. Afterward, the ZIF-8 polyhedrons were collected via centrifugation, washed with ethanol several times, then dried at 60 °C.

ZIF-8@ZIF-67 polyhedrons were prepared according to a previously reported method, with some modifications [1]. Briefly, 300 mg of $Co(NO_3)_2{\cdot}6H_2O$ and 20 mL of methanol solution containing 756 mg of 2-methylimidazole were quickly poured into 60 mL methanol solution containing 200 mg of ZIF-8, and the mixture was stirred at room temperature for 24 h. The generated purple ZIF-8@ZIF-67 particles were collected via centrifugation, washed with ethanol several times, then dried at 60 °C.

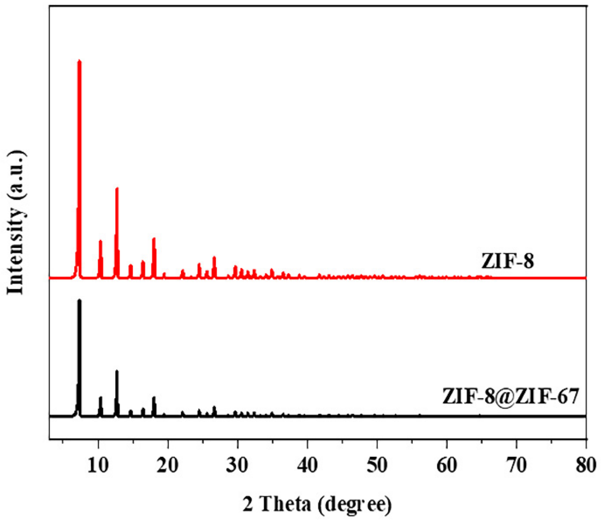

Figure S1. XRD patterns of as-prepared ZIF-8 and ZIF-8@ZIF-67.

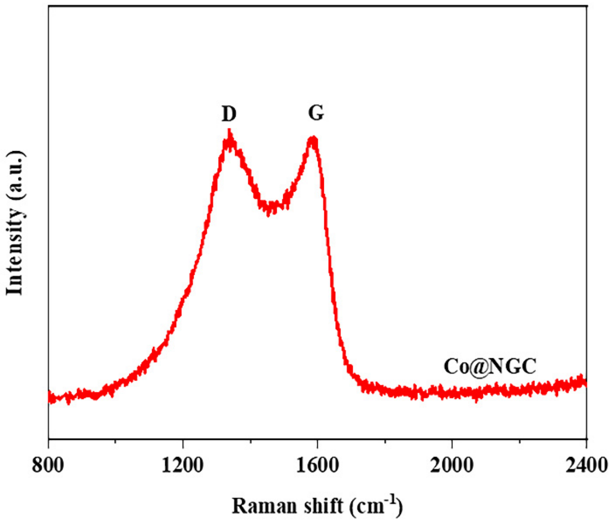

Figure S2. Raman spectrum of Co@C.

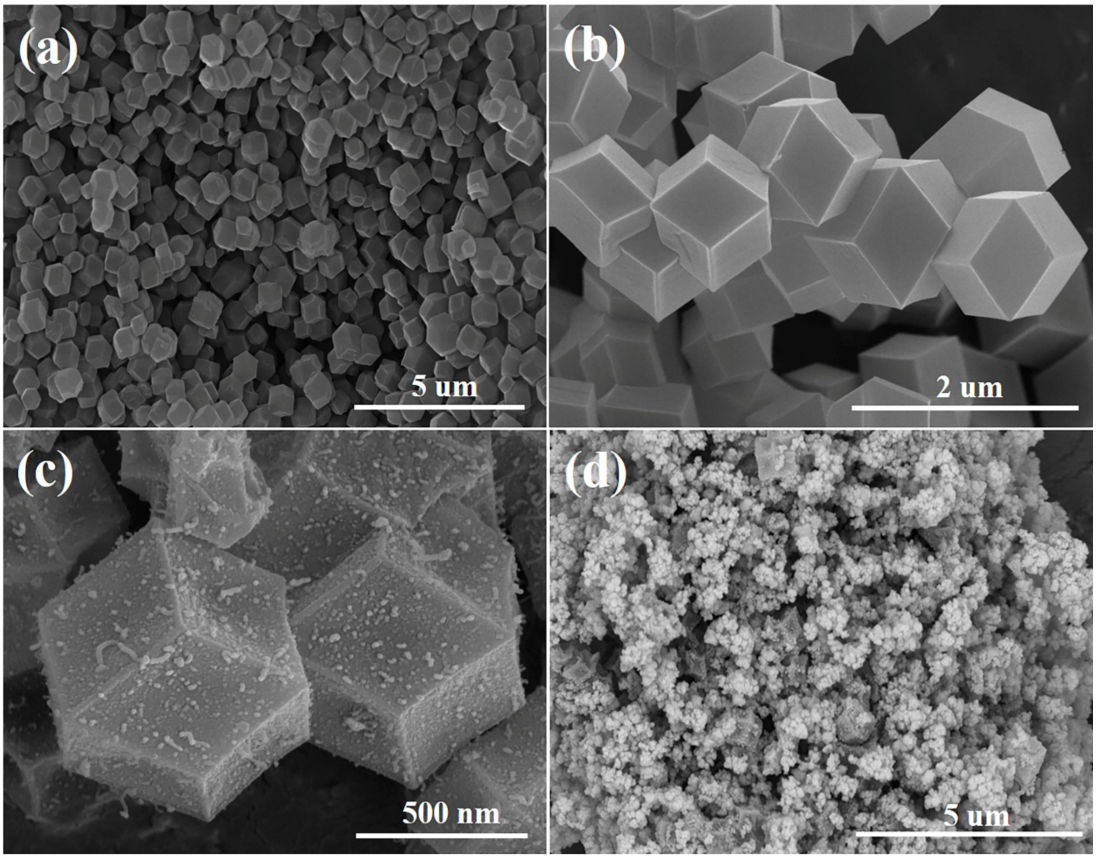

Figure S3. (a) SEM images of ZIF-8, (b) ZIF-8@ZIF-67, (c) Co@C, and (d) CdS/Co@C-7.

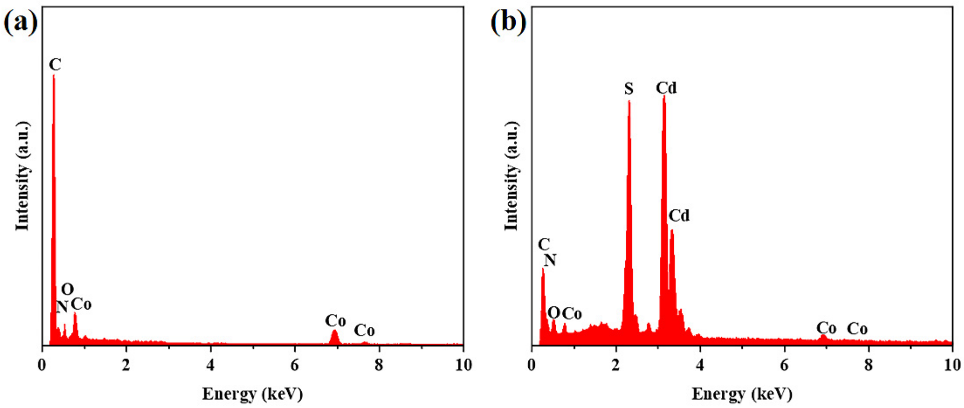

Figure S4. EDS spectra of (a) Co@C and (b) CdS/Co@C-7.

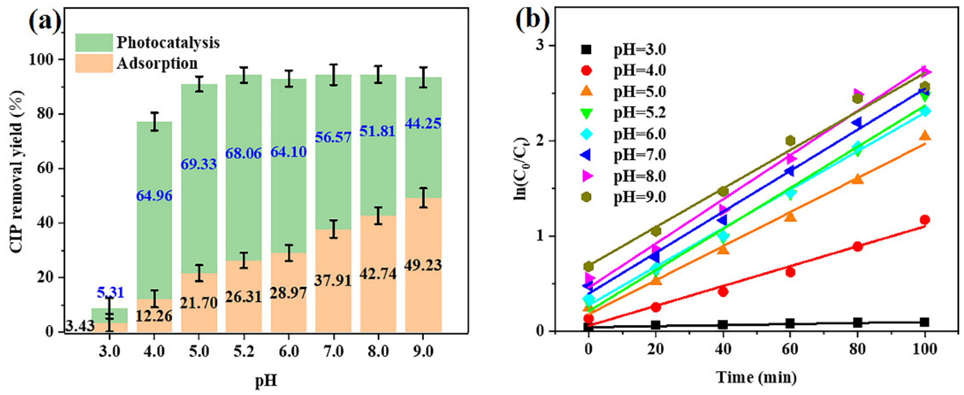

Figure S5. (a) The CIP removal yield over CdS/Co@C-7. (b) The corresponding pseudo-first-order kinetic curves for CIP photocatalytic degradation over CdS/Co@C-7.

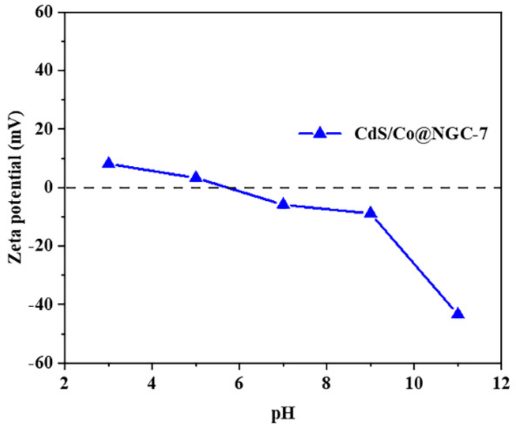

Figure S6. Variation of the Zeta potential of CdS/Co@C-7 suspended in water as a function of pH.

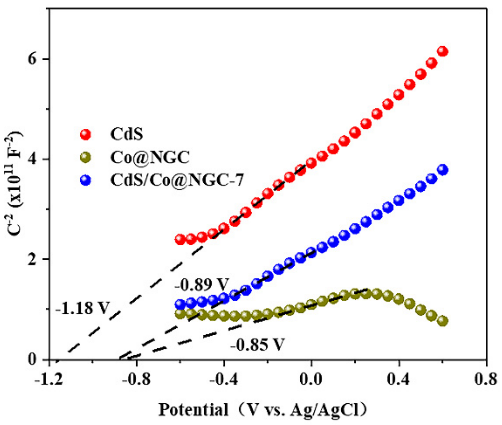

Figure S7. Mott–Schottky plots corresponding to CdS and Co@C.

Table S1. Summary of textural and optical properties of the samples.

Samples	S_{BET} (m^2•g^{-1})	Pore volume (cm^3•g^{-1})	Pore size (nm)
CdS	32.46	0.11	13.04
CdS/Co@C-1	39.10	0.12	11.15
CdS/Co@C-3	49.24	0.16	12.60
CdS/Co@C-5	53.18	0.12	11.31
CdS/Co@C-7	55.69	0.11	10.66
CdS/Co@C-10	64.06	0.14	9.96
Co@C	288.34	0.16	6.18

Table S2. The band gap and photocatalytic activities of as-prepared samples.

Samples	E_g (eV)	k (× 10^{-2} min^{-1})	R^2
CdS	2.44	0.495	0.989
CdS/Co@C-1	2.43	0.784	0.990
CdS/Co@C-3	2.43	1.095	0.988
CdS/Co@C-5	2.41	1.475	0.996
CdS/Co@C-7	2.38	2.199	0.993
CdS/Co@C-10	2.39	1.756	0.991
Co@C	0.86	0.084	0.987

References

1. Pan, Y.; Sun, K.; Liu, S.; Cao, X.; Wu, K.; Cheong, W.-C.; Chen, Z.; Wang, Y.; Li, Y.; Liu, Y., et al. Core–Shell ZIF-8@ZIF-67-Derived CoP Nanoparticle-Embedded N-Doped Carbon Nanotube Hollow Polyhedron for Efficient Overall Water Splitting. *J. Am. Chem. Soc.* **2018**, *140, (7),* 2610-2618.
